# Prostate MRI quality: clinical impact of the PI-QUAL score in prostate cancer diagnostic work-up

**DOI:** 10.1259/bjr.20211372

**Published:** 2022-02-18

**Authors:** Eleftherios Karanasios, Iztok Caglic, Jeries P. Zawaideh, Tristan Barrett

**Affiliations:** Department of Radiology, Norfolk & Norwich University Hospital, Norwich, UK; Department of Radiology, Addenbrooke’s Hospital and University of Cambridge, Cambridge, UK; Department of Radiology, Addenbrooke’s Hospital and University of Cambridge, Cambridge, UK; Department of Radiology, IRCCS Policlinico San Martino, Genoa, Italy; Department of Radiology, Addenbrooke’s Hospital and University of Cambridge, Cambridge, UK

## Abstract

**Objective:**

To assess the reproducibility and impact of prostate imaging quality (PI-QUAL) scores in a clinical cohort undergoing prostate multiparametric MRI.

**Methods:**

PI-QUAL scores were independently recorded by three radiologists (two senior, one junior). Readers also recorded whether MRI was sufficient to rule-in/out cancer and if repeat imaging was required. Inter-reader agreement was assessed using Cohen’s κ. PI-QUAL scores were further correlated to PI-RADS score, number of biopsy procedures, and need for repeat imaging.

**Results:**

Image quality was sufficient (≥PI-QUAL-3) in 237/247 (96%) and optimal (≥PI-QUAL-4) in 206/247 (83%) of males undergoing 3T-MRI. Overall PI-QUAL scores showed moderate inter-reader agreement for senior (*K* = 0.51) and junior-senior readers (*K* = 0.47), with DCE showing highest agreement (*K* = 0.47). With PI-QUAL-5 studies, the negative MRI calls increased from 50 to 87% and indeterminate PI-RADS-3 rates decreased from 31.8. to 10.4% compared to lower quality PI-QUAL-3 studies. More patients with PI-QUAL scores 1–3 underwent biopsy for negative (47%) and indeterminate probability (100%) MRIs compared to PI-QUAL score 4–5 (30 and 75%, respectively). Ability to rule-in cancer increased with PI-QUAL score, from 50% at PI-QUAL 1–2 to 90% for PI-QUAL 4–5, with a similarly, but greater effect for ruling-out cancer and at a lower threshold, from 0% for scans of PI-QUAL 1–2 to 67.1% for PI-QUAL 4 and 100% for PI-QUAL-5.

**Conclusion:**

Higher PI-QUAL scores for image quality are associated with decreased uncertainty in MRI decision-making and improved efficiency of diagnostic pathway delivery.

**Advances in knowledge:**

This study demonstrates moderate inter-reader agreement for PI-QUAL scoring and validates the score in a clinical setting, showing correlation of image quality to certainty of decision making and clinical outcomes of repeat imaging and biopsy of low-to-intermediate risk cases.

## Introduction

Prostate cancer (PCa) is the second commonest male malignancy,^1^ and MRI is now established as the first-line investigation in the diagnostic work-up of patients presenting with suspected PCa.^2,3^

High image quality is essential for optimal outcomes from the MR-directed prostate pathway, with poor quality being associated with a greater degree of uncertainty and a lower accuracy.^4–7^ The Prostate Imaging-Reporting and Data System (PI-RADS) guidelines offer advice on minimally acceptable parameters for performing prostate multiparametric MRI (mpMRI), however, application of these between centres varies and compliance alone does not guarantee sufficient image quality.^8–11^ Furthermore, image quality is vulnerable to patient-related degradations relating to bulk motion, rectal spasm, pelvic metalwork, and/or susceptibility artefact from air at the rectoprostatic interface. Being able to both recognise and quantify image quality is important to optimise outcomes, particularly as corrective measures can be applied at either a centre-level^12^ or a patient-level^13–17^ in order to directly improve performance.

Several studies have tried to measure image quality in order to gauge the success of an intervention, however, the in-house scoring systems employed typically vary in scale and in features assessed, making comparisons between studies challenging.^18^ The recent publication of the prostate imaging quality (PI-QUAL) system, offers a means of overcoming such limitations by providing a more standardised assessment approach.^19^ Initial studies have shown the scoring system to have good inter-reader reproducibility and to be useful for comparing the severity of artefacts between patients.^20–22^ However, no study to date has correlated the PI-QUAL system with outcomes in the PCa diagnostic pathway. Therefore, the aim of this study was to evaluate PI-QUAL scoring at a single centre employing a standardised MRI protocol, in order to assess the impact of individual patient-related image degradation on clinical performance.

## Methods and materials

This single-institution, retrospective study, was approved by the local ethics committee (Anonymised), with the need for informed consent waived. The study included 247 consecutive males undergoing 3 T mpMRI for suspected PCa during a 6-month period, from July 1, 2018 to December 31, 2018. Exclusion criteria included previous prostate biopsy, prior treatment for PCa, or a contraindication to gadolinium contrast.

### Magnetic resonance imaging

3 T MRI (MR750, GE Healthcare, Chicago, IL) was performed using a 32-channel phased-array body coil. Unless contraindicated, intravenous injection of hyoscine butylbromide (Buscopan, 20 mg ml^−1^, Boehringer, Germany) was administered prior to imaging to reduce peristaltic movement. In brief, imaging included axial *T*_1_ weighted fast spin echo (FSE) images of the pelvis and high-resolution *T*_2_ weighted fast recovery FSE images of the prostate in axial, coronal and sagittal planes. Axial *T*_2_ weighted imaging was performed using a 18 × 18 cm field of view (FOV); with slice thickness/gap of 3/0 mm and 0.7 × 0.5 mm in-plane resolution. Axial diffusion-weighted imaging (DWI) was conducted using a dual spin echoplanar imaging pulse sequence: 28 × 28 cm FOV, slice thickness/gap matched to T2, 2.2 × 2.2 mm in-plane resolution; six signal averages, with b-values 150, 750, 1400 s/mm^2^, with apparent diffusion coefficient (ADC) maps automatically generated. An additional matched b-2000 s/mm^2^ sequence was acquired using small FOV FOCUS^™^, with FOV 24 × 12 mm, 2.0 × 2.0 mm in-plane resolution. DCE-MRI was acquired as an axial three-dimensional fast spoiled gradient echo sequence matched to T2 with slice thickness/gap of 3/0 mm, 24 × 24 cm FOV, in-plane resolution 1.2 × 1.2 mm, following bolus injection of gadobutrol (Gadovist, Bayer HealthCare) via a power injector at a rate of 3 ml/s (dose 0.1 mmol/kg) followed by a 25 ml saline flush at 3 ml/s; injection at 28 s, temporal resolution 7 s.

### Image analysis

Images were reviewed for image quality by a junior radiologist (Anonymised) and by two senior uroradiologists with 11 years’ (Anonymised) and 6 years’ experience (Anonymised), with both reading approximately 1000 prostate MRIs per year and considered expert readers.^4,23^ Each reader was blinded to clinical details and to the original reports and independently assigned a subjective PI-QUAL score according to the quality of T2, DWI and DCE sequences using the described 5-point Likert scale^19,20^ : 1 = all three sequences below minimum diagnostic quality, 2 = only 1 sequence of acceptable diagnostic quality, 3 = at least two sequences taken together of acceptable diagnostic quality, 4 = 2 sequences taken independently of optimal diagnostic quality, 5 = all 3 sequences of optimal diagnostic quality (Supplementary Table 1). For the purposes of outcome evaluations, differences in opinion were resolved by consensus, assuming the most experienced reader’s opinion as the definitive one. Readers also subjectively recorded if each mpMRI was deemed sufficient either to rule-in or to rule-out PCa, whether the image quality was sufficient to perform accurate fusion to ultrasound for targeted biopsy, and if they would recall the patient for repeat imaging.

### Clinical correlation

PI-QUAL scores were correlated to the original MRI clinically reported PI-RADS scores, the number of biopsies performed in patients with MRIs reported as negative (PI-RADS scores 1–2) or indeterminate (PI-RADS score 3). Depending on clinical recommendation, biopsy was performed by either transrectal or transperineal approaches, using MRI-ultrasound fusion. For negative MRI examinations, decision to biopsy was based on clinical assessment, as described previously.^24^ Patients with a negative MRI and no biopsy were followed up for a minimum of 6 months and had at least one subsequent prostate-specific antigen (PSA) reading.

### Statistics

General characteristics with median and interquartile ranges (IQRs) for skewed continued variables were calculated. Pi-QUAL scores were compared with the PI-RADS scores, biopsy rate and subjective ability to rule in/out cancer. Weighted Cohen’s κ was performed, to assess inter-reader agreement, where 0–0.20 = slight, 0.21–0.40 = fair, 0.41–0.60 = moderate, 0.61–0.80 = substantial, and 0.81–1.00 = almost perfect agreement.^25^ Statistical analyses were conducted using GraphPad Prism (v. 9.0.2, GraphPad Software, San Diego, CA).

## Results

247 biopsy-naïve males with suspected PCa were assessed. Mean age was 65.7 years (median 66.9, IQR 58.8–71.3), mean PSA was 8.1 ng ml^−1^ (median 6.1, IQR 4.5–8.9), and mean prostate volume was 62.1 ml (median 54.9, IQR 45.0–81.0), with a mean PSA density of 0.16 ng/ml2 (median 0.11, IQR 0.08–0.17) (Table 1). Biopsy was performed in 141/247 men, with a cancer diagnosis confirmed in 88/247 (35.6%) and clinically significant cancer Gleason ≥3+4 in 71/247 (28.7%) patients. Distribution of PI-RADS scores in the original reports was 1–2 for 136 patients, 3 for 28 patients, 4 for 44 patients and 5 for 39 patients (Supplementary Table 2).

**Table 1.** Patient cohort data

**Age (years**)	66.9 (IQR 59–71)
**PSA value (ng/ml**)	6.1 (IQR 4.5–8.9)
**Prostate volume (ml**)	54.9 (IQR 36–81)
**PSA density (ng/ml^2^**)	0.11 (IQR 0.08–0.17)
*PSA-Density<0.10 ng/mL^2^ (n*)	*94*
*PSA-Density 0.10–0.20 ng/mL^2^ (n*)	*109*
*PSA-Density≥0.20 ng/mL^2^ (n*)	*44*
**Group 2 & 3 (Gl 3 + 4/4+3=7**)	47
**Group 4 (GS 8**)	4
**Group 5 (GS 9 and 10**)	20

GS, Gleason score; Gl, Gleason; IQR, interquartile range;PSA, prostate-specific antigen.

Age, PSA, prostate volume, and prostate density values presented as medians with Q1/Q3 values in parentheses.

### Image quality and interrater reliability

MpMRI was of at least sufficient diagnostic quality (PI-QUAL≥3) for 237/247 scans (96%) and of optimal quality (PI-QUAL≥4) for 206/247 scans (83%). Regarding the individual sequences, for *T*_2_WI 222/247 (90%) were of diagnostic quality, for DCE 222/247 (90%), and 202/247 scans (82%) for DWI. The inter-reader agreement for subjective scoring of image quality was moderate between senior readers (*K* = 0.51) and between senior and junior readers (*K* = 0.47). For the individual MRI sequences, the inter-reader agreement between senior readers was moderate (*K* = 0.41) for DCE, fair (*K* = 0.29) for DWI and slight (*K* = 0.17) for *T*_2_ weighted imaging (Table 2).

**Table 2.** Inter-reader κ agreement for PI-QUAL scores

Variable	κ	Scans (n)
Senior *vs* Junior	0.47	126
Senior *vs* Senior	0.51	50
*T*_2_WI	0.17	50
DWI	0.29	50
DCE	0.41	50

DCE, dynamic contrast-enhanced; DWI, diffusion-weighted imaging; PI-QUAL, prostate imaging quality; mpMRI, multiparametric MRI.

Agreement between senior and junior readers for overall PI-QUAL scores and senior to senior agreement for individual mpMRI sequences.

### Image quality impact on reporting of MRIs

**Figure 1.**
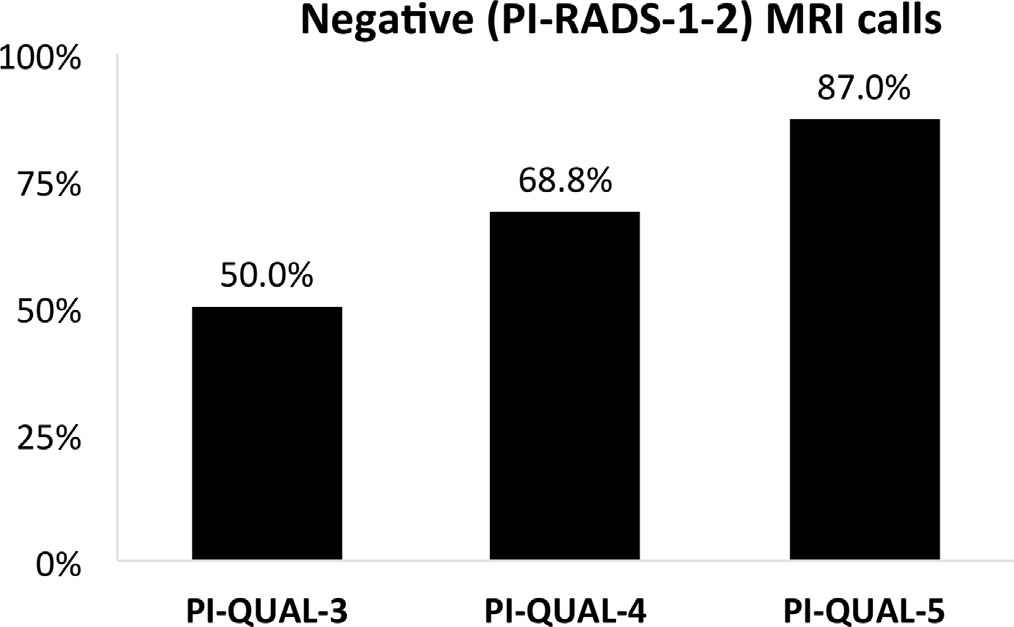
Percentage of negative mpMRIs calls (PI-RADS score 1–2) compared to PI-QUAL score. mpMRI, multiparametric MRI; PI-RADS, Prostate Imaging-Reporting and Data System; PI-QUAL, prostate imaging quality.

**Figure 2.**
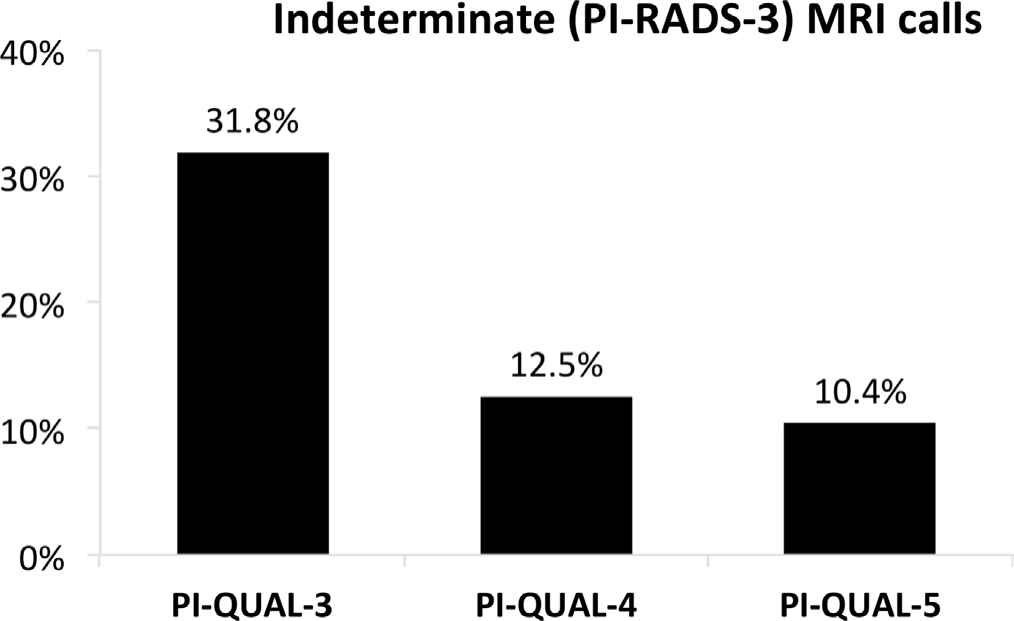
Percentage of indeterminate mpMRIs calls (PI-RADS score 3) compared to PI-QUAL score. mpMRI, multiparametric MRI; PI-RADS, Prostate Imaging-Reporting and Data System; PI-QUAL, prostate imaging quality.

The percentage of mpMRIs with negative calls (PI-RADS score 1/2) steadily increased with increasing image quality, from 50% for PI-QUAL-3, to 68.8% for PI-QUAL-4 and 87.0% for PI-QUAL-5 (Figure 1, Table 3). Additionally, the percentage of indeterminate mpMRIs (PI-RADS score 3) decreased with increasing image quality, from 31.8% for PI-QUAL-3, to 12.5% for PI-QUAL-4 and 10.4% for PI-QUAL-5 (Figure 2).

**Table 3.** MRI calls for PI-QUAL scores

Variable	PI-QUAL-3	PI-QUAL-4	PI-QUAL-5
PI-RADS 1–2	50.0%	66.8%	87.0%
PI-RADS 3	31.8%	12.5%	10.4%

Percentage of negative (PI-RADS score 1–2) and indeterminate (PI-RADS score 3) mpMRIs calls compared to PI-QUAL score.

moMRI, multiparametric MRI; PI-RADS, Prostate Imaging-Reporting and Data System; PI-QUAL, prostate imaging quality.

### Image quality impact on clinical outcomes

**Figure 3.**
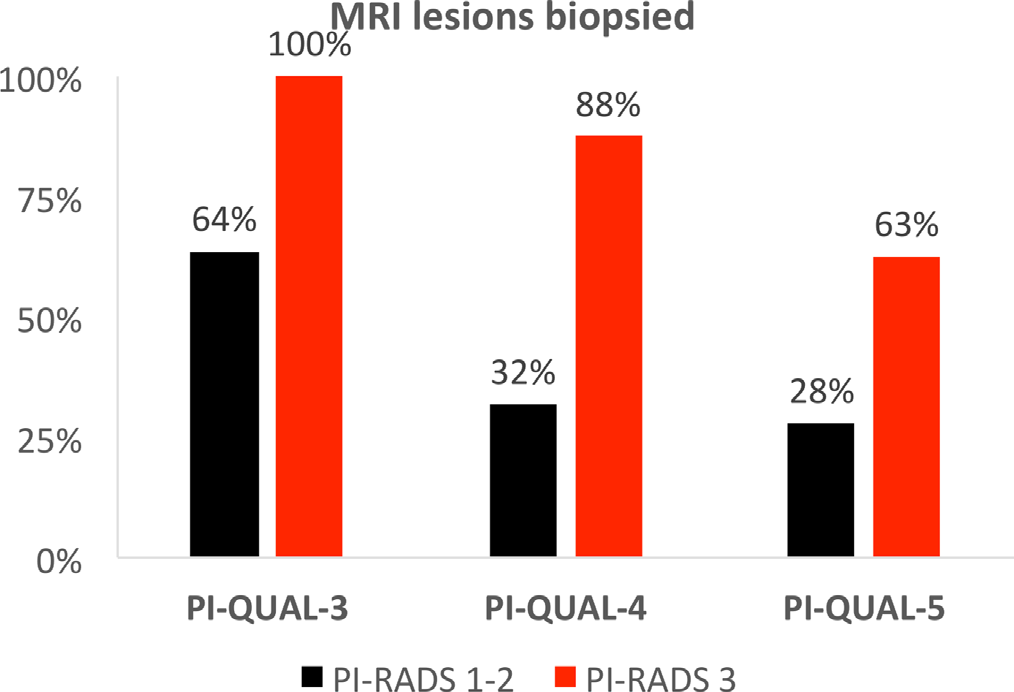
Percentage of low probability MRIs (PI-RADS-1/2 and PI-RADS-3) requiring prostate biopsy compared to PI-QUAL score. PI-RADS, Prostate Imaging-Reporting and Data System; PI-QUAL, prostate imaging quality.

The number of patients with low probability MRI studies undergoing biopsy inversely correlated with image quality, with the percentage of biopsies performed in negative MRI cases (PI-RADS score 1–2) dropping from 64% in males with MRIs of PI-QUAL score 3 to 28% for those with PI-QUAL score 5; additionally, the percentage of biopsies performed for indeterminate lesions (PI-RADS score 3) dropped from 100% for PI-QUAL 3 to 63% for PI-QUAL score 5 (Figure 3).

### Subjective assessment of clinical impact

**Figure 4.**
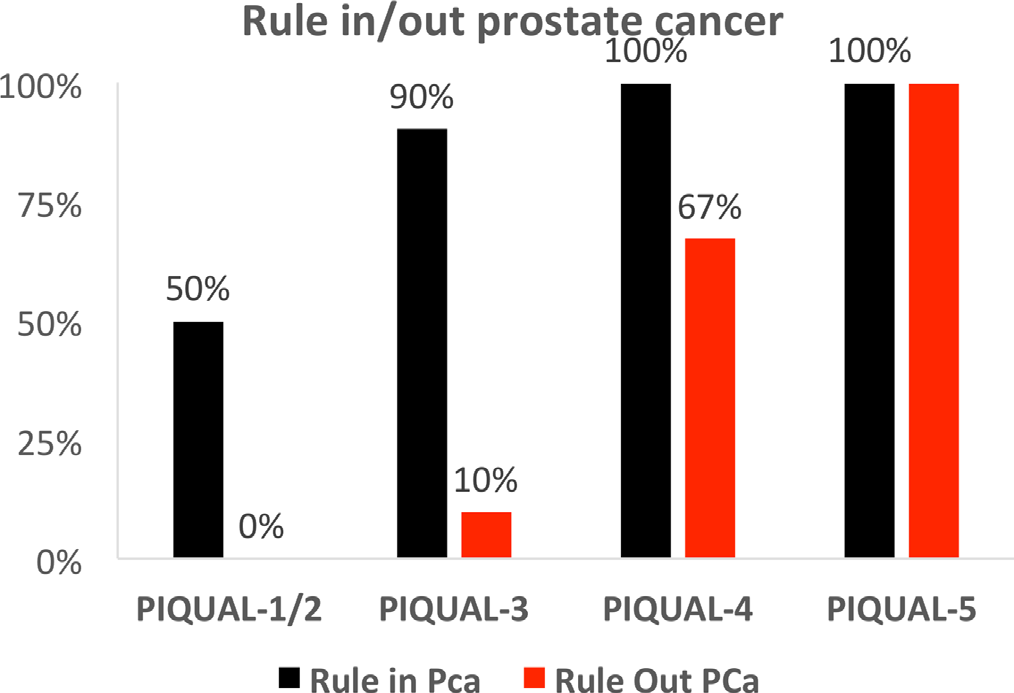
Percentage of scans sufficient to rule in and rule out prostate cancer in mpMRIs of differing quality. Percentage of scans scored by readers as being sufficient to rule in (black bars) and rule out (red bars) clinically significant prostate cancer compared to PI-QUAL score. Note the higher quality threshold required for ruling out prostate cancer compared to ruling in. mpMRI, multiparametric MRI; PI-QUAL, prostate imaging quality.

**Figure 5.**
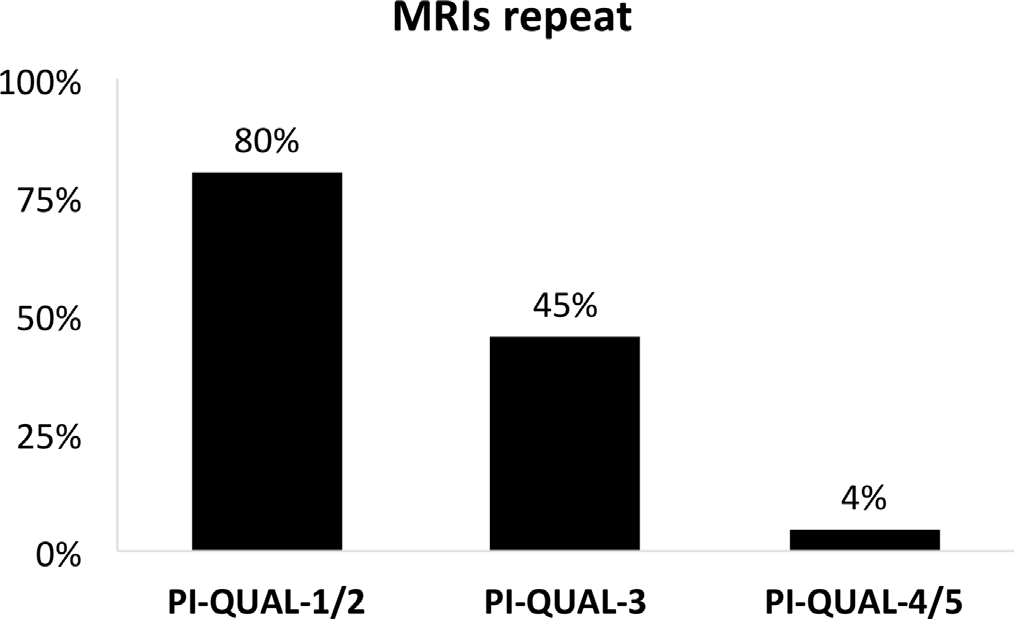
Percentage of mpMRI scans that were not considered in the new reads of diagnostic quality, and that should ideally be repeated, compared to original baseline imaging PI-QUAL score. mpMRI, multiparametric MRI; PI-QUAL, prostate imaging quality.

**Figure 6.**
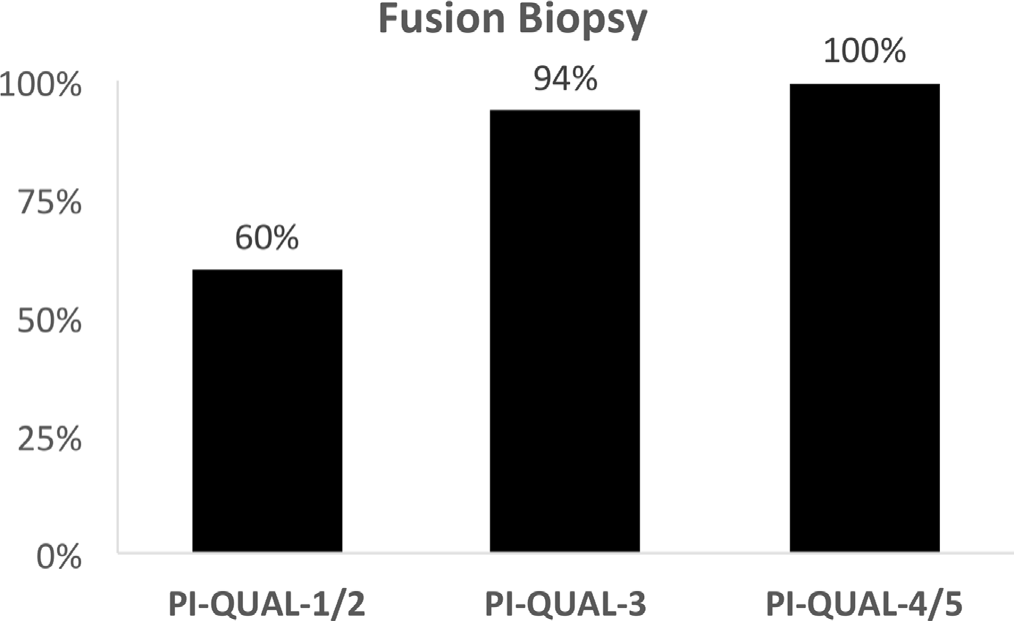
Percentage of mpMRI scans that were considered in the new reads of sufficient quality to perform fusion biopsy, compared to original baseline imaging PI-QUAL score. mpMRI, multiparametric MRI; PI-QUAL, prostate imaging quality.

The subjective decision of readers being able to rule in PCa increased with increasing image quality, from 50% of mpMRIs for PI-QUAL 1–2, to 90% for PI-QUAL 3 and 100% for PI-QUAL 4–5 (Figure 4). Similarly, the confidence of readers to rule out PCa also increased with better image quality. However, the impact of mpMRI quality was much greater on ruling out cancer, with the percentage of scans confidently ruling out cancer ranging from 0% for scans of PI-QUAL 1/2, to 10% for PI-QUAL 3, 67% for PI-QUAL 4 and 100% for PI-QUAL-5 (Figure 4). MpMRIs with higher PI-QUAL scores for image quality were subjectively scored as less likely to be repeated, with the percentage of mpMRIs that should be repeated dropping from 80% for PI-QUAL score 1/2, to 45% for PI-QUAL 3 and 4% for PI-QUAL 4/5 (Figure 5). Additionally, mpMRIs with higher PI-QUAL scores were scored as more likely to be suitable for guiding a fusion biopsy, with their percentage increasing from 60% for PI-QUAL score 1/2, to 94% for PI-QUAL 3 and 100% for PI-QUAL 4/5 (Figure 6).

## Discussion

Our single-centre study demonstrated a high overall image quality using a standardised 3 T prostate MRI protocol. We evaluated the impact of image quality on clinical outcomes and decision making, showing that the threshold to rule out cancer on a study was higher than the rule in threshold, and that a lower PI-QUAL score objectively correlated with higher number of indeterminate MRI results and increased biopsy procedures for lower probability studies (PI-RADS score 1–3).

Overall, the quality of mpMRI within our study exceeded the results from the original PI-QUAL paper which assessed 58 MRIs from 22 centres, with 60% scoring PI-QUAL ≥3 and 95% PI-QUAL ≥4, compared to 83 and 96%, respectively in our study.^19^ The result is expected as the PRECISION study was a multicentre study performed using both 1.5 T and 3 T, whereas here imaging was performed on a single 3 T scanner using a uniform protocol. Variations in image quality and PI-QUAL scores are therefore likely to be related to patient-specific factors such as motion, rectal wall spasm, contraindication to antiperistaltic agents, gas distension, or presence of pelvic metalwork. The overall inter-reader agreement was similar for senior readers (*K* = 0.51) and between junior and senior readers (*K* = 0.47), with the highest individual sequence agreement observed for DCE (*K* = 0.41). The overall agreement was notably lower than a recent study from the PI-QUAL authors (*K* = 0.82),^20^ however, our agreement is similar to a recent study published using PI-QUAL with a κ of 0.42^22^ and is in line with the interobserver agreement of PI-RADS scores in multicentre reader studies,^26–28^ and for in-house image quality scoring systems employed for other imaging studies.^29–32^

**Figure 7.**
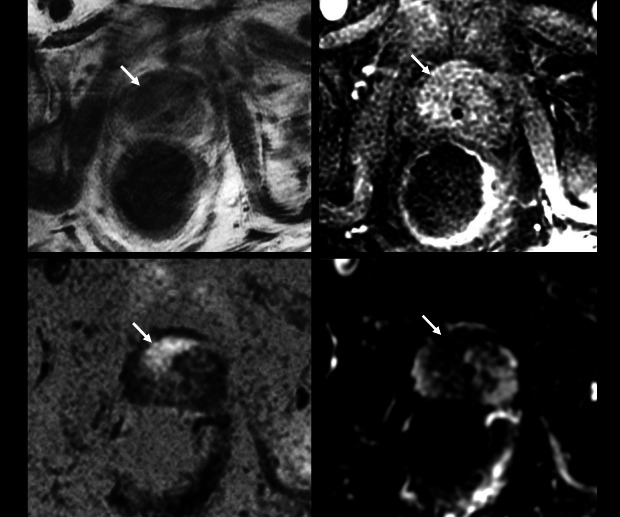
Example of poor-quality study sufficient to rule-in prostate cancer. 74 y/o, PSA 13.19 ng ml^−1^. Significant motion artefact (bulk motion and rectal spasm) affects the study. T2 (**A**), b-2000 DWI (**B**) and ADC map (**C**), and DCE (**D**) were all scored “no” for diagnostic quality. PI-QUAL Score 1/5. There is however, a clear 20 × 16 mm anterior right mid TZ lesion (arrows), minimizing the clinical impact of this poor-quality study. Target biopsy: Gleason 3 + 4=7; 25% pattern 4 in 3/3 cores (max length 10 mm). The patient was treated with androgen deprivation therapy. ADC, apparent diffusion coefficient; DWI, diffusion-weighted imaging; PSA, prostate-specific antigen; PI-QUAL, prostate imaging quality.

**Figure 8.**
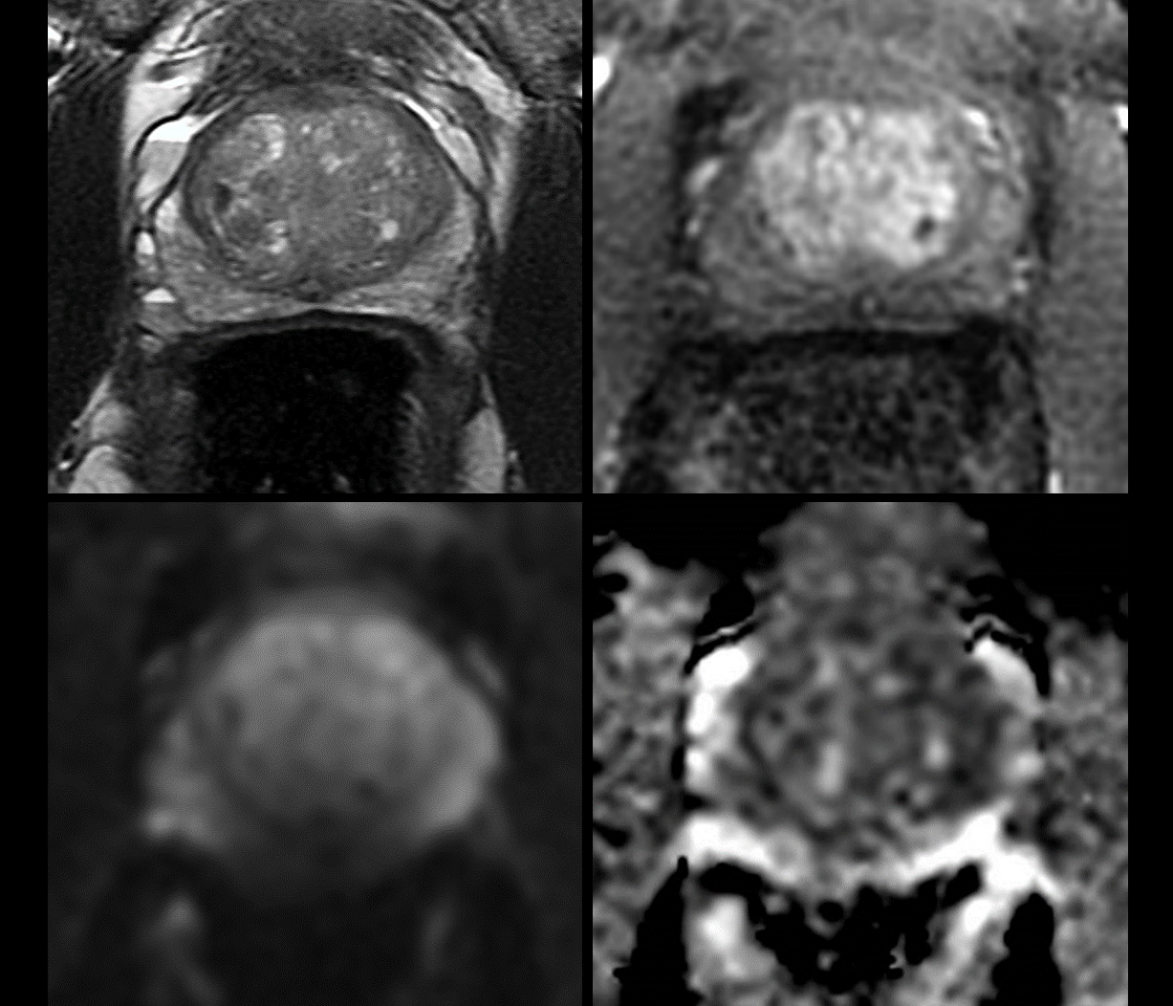
Example of sufficient quality study not sufficient to rule out prostate cancer and requiring a biopsy. 64 y/o, PSA 4.9 ng ml^−1^. (A) T2 shows some minor motion artefact. (B, C) Air at rectal–prostatic interface causes susceptibility artefact on b-1400 DWI (**B**) and ADC maps (**C**) were scored as not diagnostic. (D) DCE was independently of diagnostic quality. T2 and DCE combined were deemed of acceptable quality with overall PI-QUAL Score 3/5. Theoretically at this score threshold, clinically significant cancer can be both ruled-in and ruled-out, however, as DWI is the dominant PZ sequence, both readers recorded that csPCa could not be ruled out. In this case, TRUS biopsy was performed (all 12 cores negative). ADC, apparent diffusion coefficient; csPCa, clinically significant prostate cancer; DWI, diffusion-weighted imaging; PSA, prostate-specific antigen; PI-QUAL, prostate imaging quality

Lower PI-QUAL scores and poorer image quality was associated with increased uncertainty in the MRI decision-making, with a higher call rate of PI-RADS score three with PI-QUAL score 1–3 studies compared to those with PI-QUAL score 4–5. This is further supported by the increasing trend for calling a negative MRI when the image quality was PI-QUAL 4–5. Higher image quality also allowed for greater subjective confidence in being about to both exclude and confirm tumours, supporting the PI-QUAL implications of a score 3, which can rule-in, but not rule-out cancer, and highlighting that clinical impact does not always directly relate to image quality. This differing threshold is intuitive as larger lesions with restricted diffusion (particularly when PI-RADS score 5) are likely to be apparent even in a low quality study (Figure 7), however, a similar study would not afford the reader sufficient confidence for excluding small lesions. Although the data collection was retrospective, there is the implication that image quality also affected clinical outcomes, with more patients with negative or indeterminate MRI (PI-RADS score 1–3) undergoing biopsy at a lower PI-QUAL score (1–3) compared to higher (4–5); Figure 8. Caglic et al in a study predating the PI-QUAL recommendations also highlighted a trend towards higher biopsy numbers and with lower cancer yield in patients with increasing rectal distension and associated lower quality DWI.^33^ However, it should be noted that data relating to the clinical impact of poor image quality is limited in the existing literature, which is in contradistinction to the number of studies assessing interventions to improve image quality.

Subjectively, readers recommended a substantially higher amount of PI-QUAL score 1–2 studies (80.0%) should be repeated compared to those with higher PI-QUAL scores 4–5 (4.4%), implying the “rule-out” threshold was not met. Importantly, the recommendation to repeat a study should be informed on the likelihood of a repeat study being diagnostic, which may not be certain in patients with hip metalwork or claustrophobia/anxiety. In cases of doubt, clinical discussion in a multidisciplinary team meeting may be more appropriate, in particular regarding other clinical factors such as PSA, PSA-density, digital rectal examination and family history, which may warrant systematic biopsy regardless. Index lesion detection is not the only purpose of a prostate MRI study, and ideally, the study will be of sufficient quality to exclude smaller secondary lesions in patients that are potential candidates for either active surveillance or focal therapy selection, enable planning and fusion of images at the time of biopsy, and allow for accurate staging. Clearly, such “clinical impact” factors need to be considered alongside image quality scoring and MRI findings, and are likely to be addressed in future iterations of the PI-QUAL recommendations.^18^

Our study has several limitations, including a retrospective analysis. This was a single centre study using a single 3 T scanner with a standardised protocol, however, this design enabled evaluation of clinical outcomes related to patient-specific image quality factors. Our MRI protocol was not fully PI-RADS compliant based on the T2 in-plane resolution of 0.7 × 0.5 mm (suggested ≥0.7×0.4 mm). Although this would theoretically exclude a PI-QUAL score 5, in line with previously published studies,^21,22^ we considered T2, DWI and DCE to pass technical requirements in order to allow a full range of PI-QUAL scoring given the study focused on patient-related effects on image quality and outcomes. The true negative rate cannot be fully established for males not undergoing biopsy, however, all males underwent a minimum of 6 months of clinical follow-up. The number of increased biopsy events in patients with lower probability MRI scores (PI-RADS 1–3) was employed as an indirect surrogate measure, and decision to biopsy will be influenced by other clinical factors such as PSA, PSA-density, or family history, however, these would be expected to be similar across the cohort.

In conclusion, we have validated features of PI-QUAL within a clinical cohort and demonstrated the clinical impact of prostate MR image quality in diagnostic work-up, with higher image quality being associated with decreased uncertainty in decision making and improved efficiency of pathway delivery.

## Supplementary Material

bjr.20211372.suppl-01

bjr.20211372.suppl-02
