# Supplementary material for: Prostate MRI quality: clinical impact of the PI-QUAL score in prostate cancer diagnostic work-up
Source: Br J Radiol. 2022 Feb 18;95(1133):20211372. doi: 10.1259/bjr.20211372 (PMC10993954; doi:10.1259/bjr.20211372)
Supplement: bjr.20211372.suppl-01 [file bjr.20211372.suppl-01.docx]

| **PI-QUAL** | **Quality of T2 / DWI / DCE sequences** | **Clinical Implications** |
| --- | --- | --- |
| **1** | All 3 below minimum diagnostic quality | It is NOT possible to rule in all significant lesions  It is NOT possible to rule out all significant lesions |
| **2** | Only 1 of acceptable diagnostic quality |  |
| **3** | ≥2 taken together of acceptable diagnostic quality | It is possible to rule in all significant lesions  It is NOT possible to rule out all significant lesions |
| **4** | 2 taken independently of optimal diagnostic quality | It is possible to rule in all significant lesions  It is possible to rule out all significant lesions |
| **5** | All 3 of optimal diagnostic quality |  |

**Supplemental Table 1.** Prostate Imaging Quality (PI-QUAL) scoring (from [19]).
