# Supplementary material for: Prostate MRI quality: clinical impact of the PI-QUAL score in prostate cancer diagnostic work-up
Source: Br J Radiol. 2022 Feb 18;95(1133):20211372. doi: 10.1259/bjr.20211372 (PMC10993954; doi:10.1259/bjr.20211372)
Supplement: Supplementary file 2 [file bjr.20211372.suppl-02.odt]

| PI-RADS score | Reports (n) |
| --- | --- |
| 1-2 | 136 |
| 3 | 28 |
| 4 | 44 |
| 5 | 39 |

Supplemental Table 2. PI-RADS scores spread in original reports.
